# Utilizing topological indices in QSPR modeling to identify non-cancer medications with potential anti-cancer properties: a promising strategy for drug repurposing

**DOI:** 10.3389/fchem.2024.1410882

**Published:** 2024-08-08

**Authors:** Shamaila Yousaf, Komal Shahzadi

**Affiliations:** Department of Mathematics, University of Gujrat, Gujrat, Pakistan

**Keywords:** topological indices, QSPR models, regression model, anti-cancer activity, non-cancer medications

## Abstract

The exploration of non-cancer medications with potential anti-cancer activity offers a promising avenue for drug repurposing, accelerating the development of new oncological therapies. This study employs Quantitative Structure-Property Relationship (QSPR) modeling to identify and predict the anti-cancer efficacy of various non-cancer drugs, utilizing topological indices as key descriptors. Topological indices, which capture the molecular structure’s geometric and topological characteristics, provide critical insights into the pharmacological interactions relevant to anti-cancer activity. By analyzing a comprehensive dataset of non-cancer medications, this research establishes robust QSPR models that correlate topological indices with anti-cancer activity. The models demonstrate significant predictive power, highlighting several non-cancer drugs with potential anti-cancer properties. Further, we will use linear, quadratic and logarithmic regression to understand the structures of anti-cancer drugs and strengthen our ability to manipulate the molecular structures. The findings underscore the utility of topological indices in drug repurposing strategies and pave the way for further experimental validation and clinical trials. This integrative approach enhances our understanding of drug action mechanisms and offers a cost-effective strategy for expanding the repertoire of anti-cancer agents.

## 1 Introduction

Cancer is a disease characterized by the uncontrolled growth and spread of certain body cells to other regions. It can originate in nearly any part of the human body, which consists of trillions of cells. Under normal circumstances, human cells grow and divide through cell division to create new cells as needed. When cells become old or damaged, they die and are replaced by new cells (Trichopoulos et al., 1996). Several symptoms and indications of this disease include weight loss, lumps, irregular bleeding, and prolonged coughing. Chewing tobacco, obesity, poor food, laziness, and increased alcohol consumption (Cleeland, 2000) are the main causes of this cancerous illness. To know more about this disease explore (Bailar and Gornik, 1997).

The rapid advancement of cancer research continually emphasizes the urgent need for new and effective oncological therapies. Traditional drug development processes are often time-consuming and expensive, prompting the scientific community to explore alternative strategies, such as drug repurposing. Drug repurposing involves identifying new therapeutic uses for existing medications, offering a cost-effective and expedited pathway to discovering anti-cancer agents (Figuerola and Avila, 2019; Gao et al., 2016; Kumar et al., 2015). This approach leverages the established safety profiles and pharmacokinetic properties of non-cancer drugs, significantly reducing the time and resources required for drug development.

In this context, Quantitative Structure-Property Relationship (QSPR) modeling emerges as a powerful tool. QSPR models utilize mathematical relationships to correlate chemical structure with biological activity, aiding in the prediction of a compound’s therapeutic potential. Specifically, topological indices, which are numerical representations capturing the geometric and topological characteristics of molecular structures, play a crucial role in these models. Topological indices have five main types: matching, mixed, eigenvalue, degree, and distance (Shanmukha et al., 2020). Degree-based topological indices on anti-cancer drugs are presented in this paper. Topological indices provide valuable insights into the molecular features that influence drug activity, making them indispensable in the identification of potential anti-cancer properties of non-cancer medications. For more details of QSPR analysis, see (Ren, 1999; Havare, 2021; Zhong et al., 2021; Adnan et al., 2022; Arockiaraj et al., 2023a; Arockiaraj et al., 2023b; Arockiaraj et al., 2023c; Zaman et al., 2023).

**FIGURE 1:**
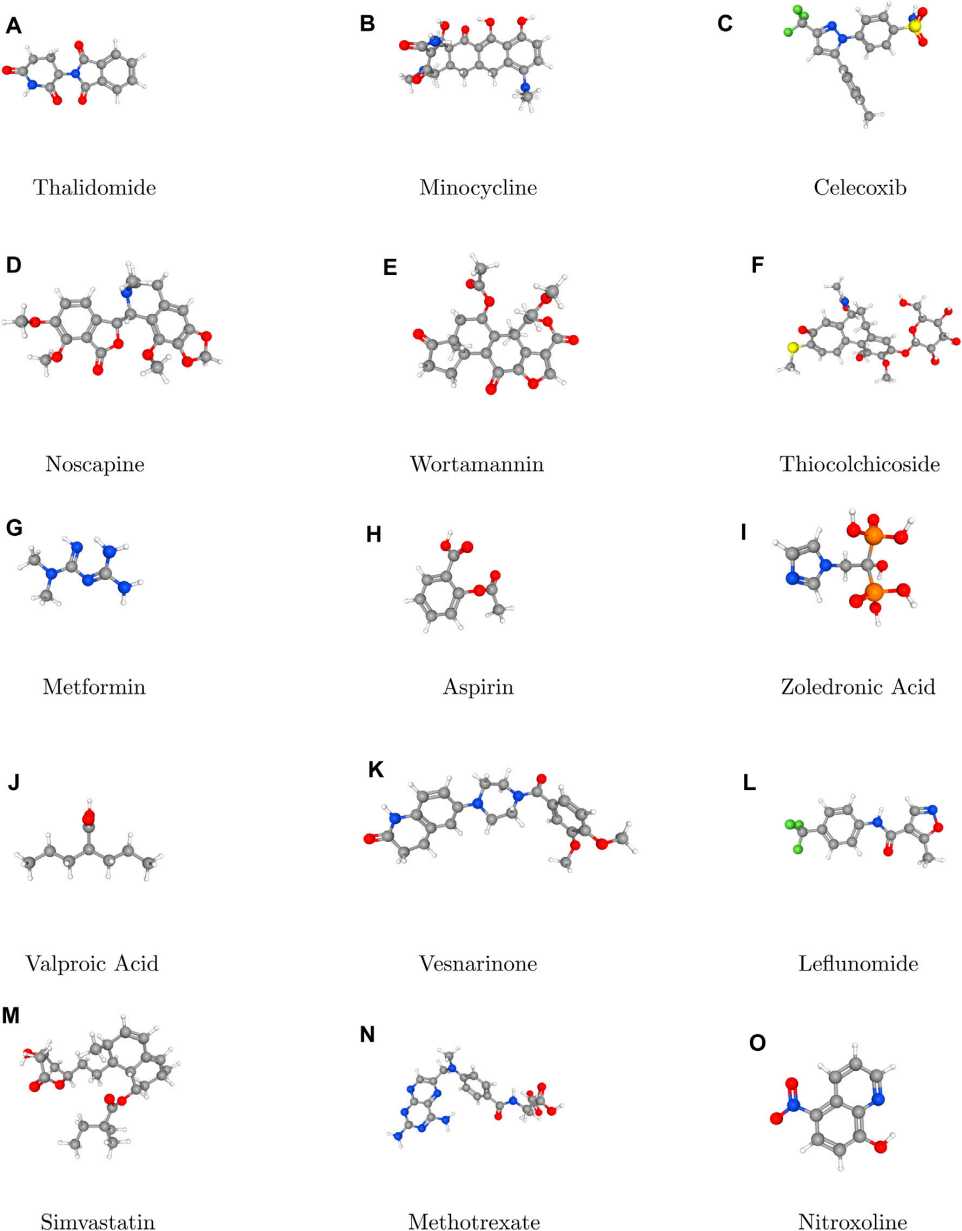
3D Structures **(A–O)** of Anti-cancer drugs.

In topological analysis, stereochemistry refers to the study of the spatial arrangements of atoms within a molecule without considering their specific three dimensional orientation. Topological analysis techniques allow chemists to understand the connectivity of atoms and the overall shape of molecules which is crucial for predicting their chemical behavior and reactivity. Topological analysis focuses on understanding how atoms are connected within a molecule. Different types of bonds (single, double and triple) and their connectivity provide information about the stereochemistry of the molecules. For example, the presence of double bonds can lead to geometric isomerism, where the spatial arrangement of substituents around the double bond affects the molecule’s properties. By analyzing the connectivity of atom and their realative positions, topological analysis can distinguish between different stereoisomeric forms of a molecule. Overall, topological analysis provides valuable insight into the stereochemistry of molecules by focusing on their connectivity, symmetric, and spatial arrangement. By considering these factors, chmeists can predict the behavior of molecules in various chemical reactions and design new compounds with desired properties. Thus, Figure 1 plays a pivotal role in elucidating the stereochemical aspects of the molecules under consideration.

This study focuses on the application of QSPR modeling, with an emphasis on topological indices, to predict and identify non-cancer drugs that may exhibit anti-cancer activity. By analyzing a comprehensive dataset of non-cancer medications, we aim to establish robust QSPR models that accurately correlate topological indices with anti-cancer efficacy. The predictive power of these models is expected to highlight several non-cancer drugs as promising candidates for further experimental validation and clinical trials. Some of the common anti-cancer drugs approved by the Food and Drug Administration (FDA) and their molecular targets are shown in Table 1. Explore (Gupta et al., 2013; D Amato et al., 1994; Gasic et al., 1972; Michaelis et al., 2007; Jendrossek, 2013; Jiang et al., 2018; Wang et al., 2010; Zekri et al., 2014; Rezaei et al., 2023) to learn about these anti-cancer drugs. Drugs were selected based on a meticulous assessment encompassing structural characteristics, predicted anti-cancer activity, and suitability for repurposing. Leveraging topological indices and quantitative structure-property relationship (QSPR) modeling, we prioritized molecules with favorable molecular connectivity patterns indicative of potential anti-cancer properties. Each selected drug underwent scrutiny for safety profiles, availability, known mechanisms of action, and previous clinical data, ensuring a comprehensive approach towards identifying promising candidates for repurposing. This methodical selection process aimed to maximize the likelihood of uncovering non-cancer medications with significant potential for anti-cancer therapeutic applications (Singhal et al., 2022). The implications of this research are significant. Not only does it enhance our understanding of the molecular mechanisms underlying drug action, but it also paves the way for more efficient and cost-effective drug discovery processes. By integrating computational modeling with drug repurposing strategies, this study contributes to expanding the repertoire of available anti-cancer agents, ultimately improving therapeutic outcomes for cancer patients.

**TABLE 1:** Non-cancer drugs and their mechanism of action for non-cancer and cancer activities.

*Drug*	*Original indication*	*New anti-cancer indication*
*Thalidomide*	*Antiemetic during gestation*	*Multiple myeloma*
*Aspirin*	*Analgesic, antipyretic*	*Colorectal cancer*
*Valproic Acid*	*Antiepileptic*	*Leukemia, solid tumors*
*Celecoxib*	*Osteoarthritis, rheumatoid arthritis*	*Colorectal cancer, lung cancer*
*Leflunomide*	*Rheumatoid arthritis*	*Prostate cancer*
*Wortmannin*	*Antifungal*	*Leukemia*
*Zoledronic Acid*	*Anti-resorption of bone*	*Muliple myeloma, prostate cancer*
*Minocycline*	*Acne*	*Ovarian cancer, glioma*
*Metformin*	*Diabetes mellitus*	*Breast, Prostate, colorectal*
*Thiocolchicoside*	*Muscle relaxant*	*Leukemia, muliple myeloma*
*Noscapine*	*Antitussive, antimalarial*	*Mupltiple cancer types*
*Nitroxoline*	*Antibiotic*	*Bladder, breast cancer*
*Methotrexate*	*Acute leukemia*	*Osteosarcoma, breast cancer*
*Vesnarinone*	*Cardioprotective*	*Oral cancer, lymphoma*
*Simvastatin*	*Hyperlipidemia*	*Pituitary neuroendocrine tumors*

Consider G (V,E) a molecular graph with vertex and edge sets denoted by V and E, respectively. The number of vertices adjacent to a vertex is known as the degree of a vertex which is denoted as deg(v), while d (u,v) is the shortest distance between two vertices. In this work, we will use following topological indices.

### 1.1 Difference between ABC and R index ABC-R(G)

In present times, there is a growing interest in studying the correlation or comparison between topological indices, see (Das et al., 2016). Recently, Ali and Du (Ali and Du, 2017) explored extremal binary and chemical trees, specifically focusing on the difference between ABC and R indices. The ABC-R index provides additional information about the molecular structure beyond just the topological complexity measured by the ABC index alone. It accounts for the size of the molecule and provides insights into its topological properties relative to its size. The ABC-R index is defined as

$$\left(ABC-R\right)\left(G\right)=\underset{s,t\in E\left(G\right)}{\sum }\frac{\sqrt{d_{s}+d_{t}-2}-1}{\sqrt{d_{s}d_{t}}}.$$

### 1.2 Geometric arithmetic index GA(G)

Another recently conceived vertex-degree-based topological index utilizes the difference between the geometric and arithmetic means and is defined as

$$GA\left(G\right)=\underset{s,t\in E\left(G\right)}{\sum }\frac{2\sqrt{d_{s}d_{t}}}{d_{s}+d_{t}}.$$

Where, of course $\sqrt{d_{s}d_{t}}$ and $\frac{1}{2}\left(d_{s}+d_{t}\right)$ are the geometric and arithmetic means, respectively, of the degrees of the end-vertices of an edge.The “geometric-arithmetic index,” devised by Vuki $\overset{ˇ}{c}$ evi $\overset{´}{c}$ and Furtula (Vukicevic and Furtula, 2009), has garnered attention not just for its mathematical investigation but also for its practical applications in chemistry. Particularly noteworthy to chemists are its applications in analyzing acyclic, unicyclic, and bicyclic molecular graphs (Du et al., 2011), as well as in studying benzenoid hydrocarbons and phenylenes.

### 1.3 Multiplicative first exponential Zagreb index $\Pi_{1}$ (G)

The multiplicative first exponential Zagreb index (Akgunes and Aydin, 2021) is a molecular descriptor in chemical graph theory. This index provides valuable structural information about the molecular graph, with a focus on the significance of highly connected vertices. This index is used in quantitative structure-property relationship (QSPR) studies to predict various chemical and physical properties of molecules. These properties may include boiling points, solubility, stability, and other characteristics relevant in chemistry and pharmacology.

$$E\Pi_{1}\left(G\right)=\underset{t\in V\left(G\right)}{\prod }e^{d_{t}^{2}}.$$

### 1.4 Multiplicative second exponential Zagreb index $\Pi_{2}$ (G)

The multiplicative second exponential Zagreb index (Akgunes and Aydin, 2021) provides valuable structural information about the molecular graph, focusing on the significance of pairs of highly connected vertices. It is used to predict various chemical and physical properties of molecules, particularly emphasizing the role of vertex degrees and their pairwise interactions in determining molecular behavior.

$$E\Pi_{2}\left(G\right)=\underset{t\in V\left(G\right)}{\prod }e^{d_{s}d_{t}}.$$

### 1.5 Multiplicative geometric arithmetic index GA Π (G)

Multiplicative GA index (Kulli, 2016) is a molecular descriptor that is calculated by takin the product of edge multiplicities and the geometric mean of vertex degrees in a molecular graph. This index provides information about the atom-bond connectivity in the molecular graph, considering both the geometric and arithmetic means of the degrees of the connected vertices.

$$GA\Pi \left(G\right)=\underset{s,t\in E\left(G\right)}{\prod }\frac{2\sqrt{d_{s}d_{t}}}{d_{s}+d_{t}}.$$

### 1.6 Symmetric division degree index SDD(G)

Several years ago, D. Vuki $\overset{ˇ}{c}$ evi $\overset{´}{c}$ and Ga $\overset{ˇ}{s}$ perov (Vukičević and Gašperov, 2010) considered a novel category of molecular descriptors comprising one hundred and forty-eight descriptors known as “discrete Adriatic indices.” These were proposed to enhance various QSPR/QSAR (quantitative structure-property/activity relationship) studies. However, their findings indicated that only a select few descriptors from this class proved to be beneficial. One such valuable descriptor is the symmetric division deg (SDD) index. It measures the degree of symmetry in a graph. It’s calculated as the sum of the squares of the degrees of adjacent vertices, divided by the product of their degrees, summed over all edges in the graph.

$$SDD\left(G\right)=\underset{s,t\in E\left(G\right)}{\sum }\frac{d_{s}^{2}+d_{t}^{2}}{d_{s}d_{t}}.$$

## 2 Methodology

The methodology for this study involves a systematic approach to identify and predict the anti-cancer potential of non-cancer medications using Quantitative Structure-Property Relationship (QSPR) modeling with topological indices. The process begins with data collection, where a comprehensive dataset of non-cancer medications is compiled from publicly available databases such as PubChem and ChemSpider. This dataset includes detailed molecular structures and known pharmacological properties of each drug. Next, molecular descriptor calculation is performed, where various topological indices are calculated for each drug in the dataset. These indices include the symmetric division degree index, geometric arithmetic index, multiplicative first exponential Zagreb index, difference between ABC and R index, multiplicative second exponential Zagreb index, and multiplicative geometric arithmetic index.

In the model construction phase, multiple QSPR models are developed using linear, quadratic, and logarithmic regression algorithms with the aid of SPSS software. Each type of regression model is built to explore different potential relationships between the topological indices and anti-cancer activity. Linear regression models assume a direct proportional relationship, quadratic regression models consider a parabolic relationship, and logarithmic regression models account for situations where the change in anti-cancer activity diminishes as the value of the topological indices increases. These models are trained on the selected topological indices and known anti-cancer activity data.

**FIGURE 2:**
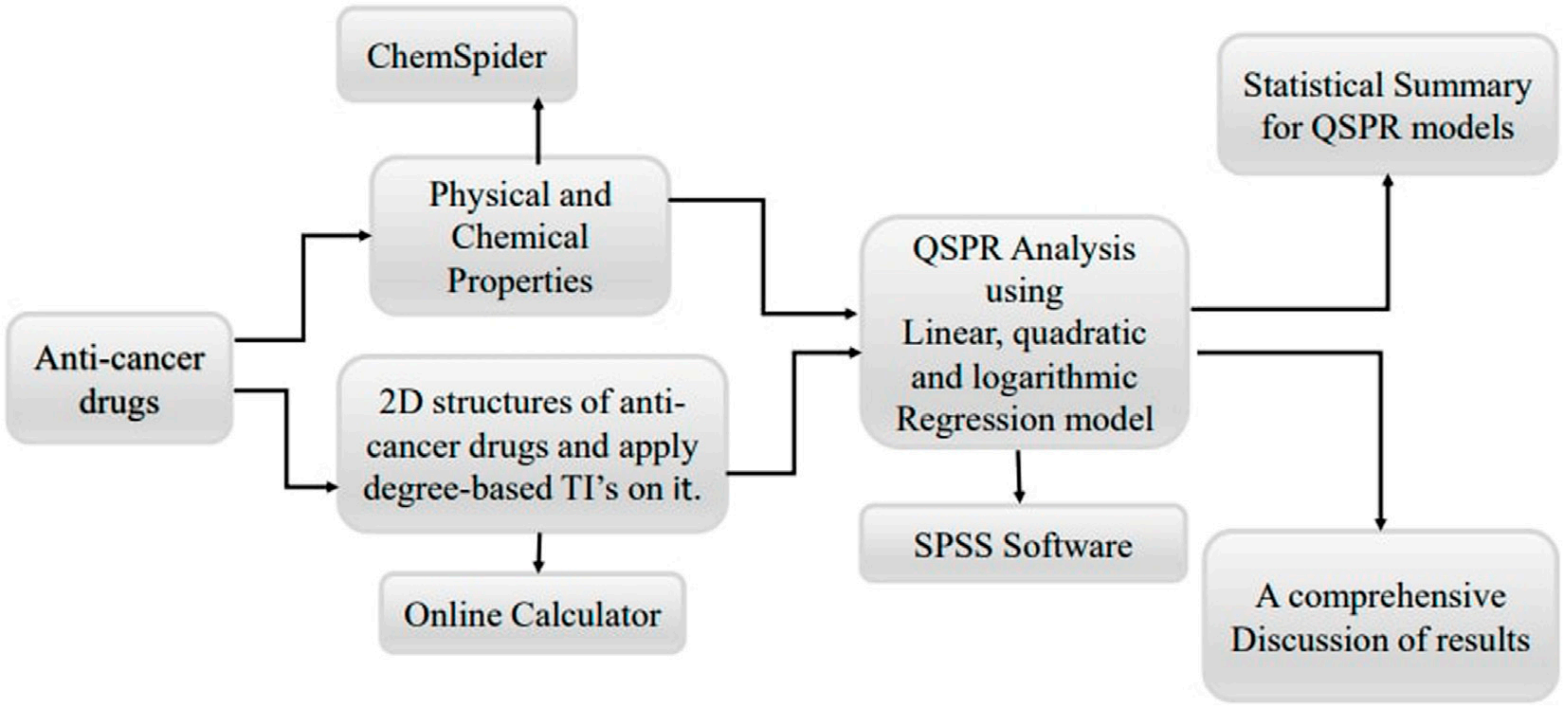
Graphical abstract.

Finally, data analysis and interpretation are performed. The experimental data are analyzed using statistical methods to compare the predicted and observed anti-cancer activities. Correlation coefficients and significance tests are used to assess the accuracy and reliability of the QSPR models. Based on the experimental validation results, the QSPR models are refined and retrained to improve their predictive power and reliability. By following this structured methodology, the study aims to establish a reliable framework for repurposing non-cancer medications as potential anti-cancer agents, leveraging the power of QSPR modeling and topological indices. Figure 2 shows the basic flowchart of this work.

## 3 Analyzing molecular structures and computing topological indices for various anti-cancer medications

**FIGURE 3:**
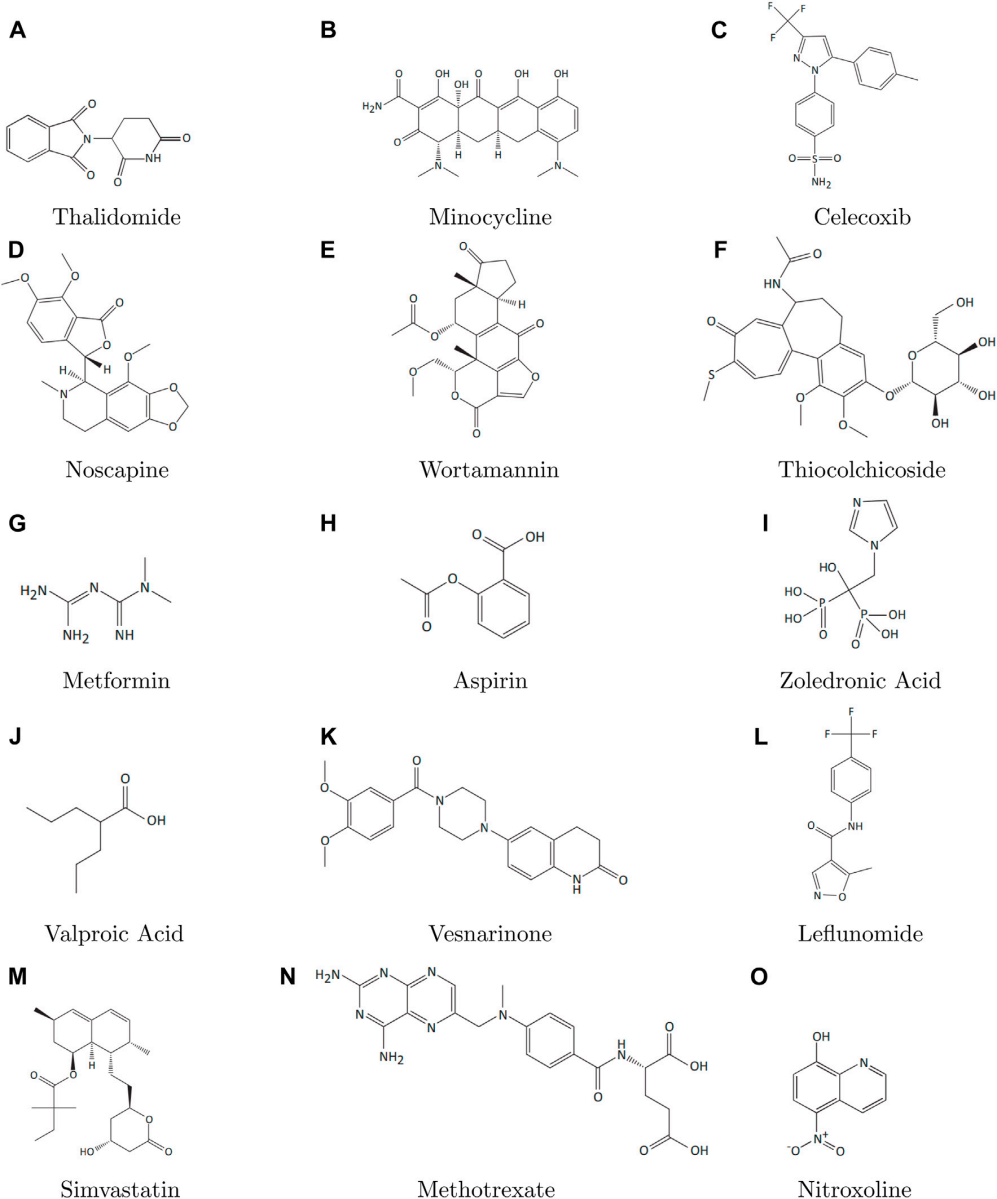
2D structures **(A–O)** of anti-cancer drugs.

Topological indices are used to analyze the different structures. In this article, six TIs are used to describe the structures of fifteen anti-cancer drugs. The structures of these drugs are shown in Figure 3. The six physio-chemical properties are obtained from ChemSpider which are given in Table 2 and the value of each topological index corresponding to each structure is computed with the help of their formulas, as shown in Table 3.

**TABLE 2:** The physio-chemical properties of non-cancer medication to be used in the treatment of cancer.

*Structures*	*MP*	*BP*	*WS*	*D*	*VP*	*MW*
*Units*	*°C*	*°C*	*mg/mL*	*g/cm^3^*	*mmHg*	*g/mol*
*Thalidomide*	*270*	*509.7*	*0.012*	*1.6*	*1.3*	*258.3*
*Aspirin*	*136*	*140*	*3*	*1.40*	*0.7*	*180.16*
*Valproic Acid*	*120*	*222*	*1.3*	*0.9*	*0.9*	*144.21*
*Celecoxib*	*158*	*529*	*3.3*	*1.43*	*1.4*	*381.372*
*Leflunomide*	*165*	*289.3*	*21*	*1.392*	*0.6*	*270.207*
*Wortmannin*	*237.52*	*615.6*	*0.121*	*1.4*	*1.8*	*428.43*
*Zoledronic Acid*	*193*	*764*	*2*	*2.13*	*2.7*	*272.09*
*Minocycline*	*213*	*803.3*	*50*	*1.6*	*0.3*	*457.28*
*Metformin*	*223*	*224.1*	*300*	*1.3*	*1.3*	*129.16*
*Thiocolchicoside*	*190*	*929.6*	*10*	*1.2222*	*0.3*	*563.618*
*Noscapine*	*174*	*565.3*	*0.181*	*1.395*	*1.5*	*413.42*
*Nitroxoline*	*180*	*385.64*	*2.73*	*1.3907*	*1.0*	*190.16*
*Methotrexate*	*212*	*823*	*2600*	*1.5*	*0.0000*	*454.4*
*Vesnarinone*	*238.1*	*678.3*	*0.0968*	*1.2*	*2.1*	*395.5*
*Simvastatin*	*135*	*564.9*	*0.0013*	*1.1*	*3.5*	*418.56*

**TABLE 3:** The values of topological indices of molecular structures of non-cancer medication to be used in the treatment of cancer.

*Structures*	*ABC-R*	*GA*	*EΠ_1_*	*EΠ_2_*	*GAΠ*	*SDD*
*Thalidomide*	*5.9115*	*2.3429*	*1467*	*1430*	*570.213*	*48.3333*
*Aspirin*	*3.44*	*12.3833*	*1420*	*4.607*	*81.4590*	*32*
*Valproic Acid*	*1.8235*	*5.2314*	*3.1856*	*8.659 × 10^16^*	*0.8000*	*22*
*Celecoxib*	*9.2836*	*26.4031*	*4.6754 × 10^64^*	*1.2384 × 10^72^*	*1160.9528*	*73*
*Leflunomide*	*5.8192*	*18.9602*	*2.2353 × 10^37^*	*4.3750 × 10^48^*	*257.9914*	*50.8333*
*Wortmannin*	*10.3553*	*33.5562*	*8.1318 × 10^79^*	*8.4682 × 10^102^*	*6449.763*	*84.3333*
*Zoledronic Acid*	*5.1584*	*13.4822*	*3.0251 × 10^36^*	*3.6379 × 10^42^*	*77.5959*	*48.75*
*Minocycline*	*11.6827*	*35.653*	*3.9452 × 10^88^*	*1.6575 × 10^114^*	*8774.3495*	*99.75*
*Metformin*	*2.1268*	*7.2897*	*4.3112 × 10^15^*	*4.3112 × 10^15^*	*8.4853*	*23*
*Thiocolchicoside*	*11.2285*	*40.6037*	*2.9152 × 10^89^*	*3.7465 × 10^108^*	*8294.384*	*97*
*Noscapine*	*9.2942*	*33.1743*	*2.7279 × 10^76^*	*4.7445 × 10^94^*	*1934.7865*	*70.75*
*Nitroxiline*	*4.0746*	*14.5173*	*1.8587 × 10^31^*	*8.2230 × 10^36^*	*162.9196*	*34.6667*
*Methotrexate*	*9.7877*	*33.5645*	*9.1511 × 10^72^*	*6.5892 × 10^83^*	*2443.7562*	*83.6667*
*Vesnarinone*	*8.5599*	*31.254*	*7.6094 × 10^66^*	*2.9915 × 10^79^*	*1440.049*	*70.6667*
*Simvastatin*	*9.6039*	*31.2514*	*1.2385 × 10^72^*	*9.7793 × 10^85^*	*1146.6172*	*83.4167*

The main purpose of this section is to give the overview of data. Derived from glutamic acid, thalidomide was initially formulated in the 1950s as a sedative-hypnotic to address pregnancy-related nausea. Recent research spanning the past decade suggests that thalidomide, initially explored for its potential anti-angiogenic properties, has the ability to influence various cell signaling pathways associated with cancer. Suppose G be the molecular structure of Thalidomide which is shown in Figure 3. The molecular graph of G has 19 vertices and 21 edges. It has four edge partition which is shown in Table 4 By using the same methodology we can determine the other topological indices. Table 1 shows the original indication and new anti-cancer indications of these drugs.

$$\begin{array}{ll} •ABC-R\left(Thalidomide\right) & =\underset{s,t\in E\left(G\right)}{\sum }\frac{\sqrt{d_{s}+d_{t}-2}-1}{\sqrt{d_{s}d_{t}}}\\ & =4\left(\frac{\sqrt{1+3-2}-1}{\sqrt{\left(1\right)\left(3\right)}}\right)+4\left(\frac{\sqrt{2+2-2}-1}{\sqrt{\left(2\right)\left(2\right)}}\right)\\ & \;+6\left(\frac{\sqrt{2+3-2}-1}{\sqrt{\left(2\right)\left(3\right)}}\right)+7\left(\frac{\sqrt{3+3-2}-1}{\left(3\right)\left(3\right)}\right)\\ & =0.9566+0.8284+1.7932+2.3333\\ & =5.9115. \end{array}$$

$$\begin{array}{ll} •GA\left(Thalidomide\right) & =\underset{s,t\in E\left(G\right)}{\sum }\frac{2\sqrt{d_{s}d_{t}}}{d_{s}+d_{t}}\\ & =4\left(\frac{2\sqrt{\left(1\right)\left(3\right)}}{1+3}\right)+4\left(\frac{2\sqrt{\left(2\right)\left(2\right)}}{2+2}\right)\\ & \;+6\left(\frac{2\sqrt{\left(2\right)\left(3\right)}}{2+3}\right)+7\left(\frac{2\sqrt{\left(3\right)\left(3\right)}}{3+3}\right)\\ & =3.4641+4+5.8788+7\\ & =20.3429. \end{array}$$

$$\begin{array}{ll} •E\Pi_{1}\left(Thalidomide\right) & =\underset{t\in V\left(G\right)}{\prod }e^{d_{t}^{2}}\\ & =e^{\left(4\right){\left(1\right)}^{2}}\times e^{\left(7\right){\left(2\right)}^{2}}\times e^{\left(8\right){\left(3\right)}^{2}}\\ & =1.46766\times 10^{45}. \end{array}$$

$$\begin{array}{ll} •E\Pi_{2}\left(Thalidomide\right) & =\underset{t\in V\left(G\right)}{\prod }e^{d_{s}d_{t}}\\ & =e^{4\left(1\right)\left(3\right)}.e^{4\left(2\right)\left(2\right)}.e^{6\left(2\right)\left(3\right)}.e^{7\left(3\right)\left(3\right)}\\ & =1.43020\times 10^{55}. \end{array}$$

$$\begin{array}{ll} •GA\Pi \left(Thalidomide\right) & =\underset{s,t\in E\left(G\right)}{\prod }\frac{2\sqrt{d_{s}d_{t}}}{d_{s}+d_{t}}\\ & =4\left(\frac{2\sqrt{\left(1\right)\left(3\right)}}{1+3}\right)\times 4\left(\frac{2\sqrt{\left(2\right)\left(2\right)}}{2+2}\right)\\ & \;\times 6\left(\frac{2\sqrt{\left(2\right)\left(3\right)}}{2+3}\right)\times 7\left(\frac{2\sqrt{\left(3\right)\left(3\right)}}{3+3}\right)\\ & =3.4641\times 4\times 5.8788\times 7\\ & =570.213. \end{array}$$

$$\begin{array}{ll} •SDD\left(Thalidomide\right) & =\underset{s,t\in E\left(G\right)}{\sum }\frac{d_{s}^{2}+d_{t}^{2}}{d_{s}d_{t}}\\ & =4\frac{{\left(1\right)}^{2}+{\left(3\right)}^{2}}{1\times 3}+4\frac{{\left(2\right)}^{2}+{\left(2\right)}^{2}}{2\times 2}\\ & \;+6\frac{{\left(2\right)}^{2}+{\left(3\right)}^{2}}{2\times 3}+7\frac{{\left(3\right)}^{2}+{\left(3\right)}^{2}}{3\times 3}\\ & =13.3333+8+13+14=48.3333. \end{array}$$

**TABLE 4:** Degrees of Thalidomide structure.

(d_s_, d_t_)	Frequency
(1, 3)	4
(2, 2)	4
(2, 3)	6
(3, 3)	7

## 4 Regression models

The following equations are used to correlate the various physical properties of various non-cancer medications used for the treatment of cancer with some topological indices. We have used the following linear, quadratic and logarithmic regression models:

$$P=A+b\left[TI\right]$$ (1)

$$P=A+b\left[TI\right]+c{\left[TI\right]}^{2}$$ (2)

$$P=A+b\ln\left[TI\right]$$ (3)

In the above Equations 1–3, p is dependent variable and TI is the independent variable. Where P is physical property of drug, A is constant, b and c are the regression coefficients. The six physiochemical properties: boiling point (BP) in ° C at 760 mmHg, molecular weight (MW) in g/mol, melting point (MP) in ° C at 760 mmHg, density(D) in $g/cm^{3}$ , water solubility (WS) in mg/mL, vapour pressure (VP) in mmHg at 25 ° C.Following are the linear, quadratic and logarithmic regression models for the defined degree-based topological indices.

### 4.1 Difference between ABC and R index ABC-R(G)

$$\begin{array}{llll} & Linear\;Models: & & Logarithmic\;Models:\\ MP & =165.979+3.277\left[ABC-R\right] & MP & =151.661+20.670\ln\left[ABC-R\right]\\ BP & =96.074+60.496\left[ABC-R\right] & BP & =-70.118+328.130\ln\left[ABC-R\right]\\ D & =1.352+0.006\left[ABC-R\right] & D & =1.230+0.091\ln\left[ABC-R\right]\\ WS & =86.975-8.395\left[ABC-R\right] & WS & =132.282-57.665\ln\left[ABC-R\right]\\ MW & =50.608+38.815\left[ABC-R\right] & MW & =-50.421+207.482\ln\left[ABC-R\right]\\ VP & =0.984+0.034\left[ABC-R\right] & VP & =0.640+0.320\ln\left[ABC-R\right]\\ & Quadratic\;Models: & \\ MP & =98.874+59.410\left[ABC-R\right]+0.081{\left[ABC-R\right]}^{2} & \\ BP & =138.290+14.016\left[ABC-R\right]-0.798{\left[ABC-R\right]}^{2} & \\ D & =0.892+0.185\left[ABC-R\right]-0.13{\left[ABC-R\right]}^{2} & \\ WS & =223.388-61.303\left[ABC-R\right]+3.929{\left[ABC-R\right]}^{2} & \\ MW & =67.781+32.155\left[ABC-R\right]+0.495{\left[ABC-R\right]}^{2} & \\ VP & =-0.928+0.775\left[ABC-R\right]-0.055{\left[ABC-R\right]}^{2} & \end{array}$$

### 4.2 Multiplicative first exponential Zagreb index $E\Pi_{1}\left(G\right)$

$$\begin{array}{llll} & Linear\;Models: & & Logarithmic\;Models:\\ MP & =189.318+0.0000\left[E\Pi_{1}\right] & MP & =165.206+0.195\ln\left[E\Pi_{1}\right]\\ BP & =497.015+0.0000\left[E\Pi_{1}\right] & BP & =104.723+3.411\ln\left[E\Pi_{1}\right]\\ D & =1.409-0.0000\left[E\Pi_{1}\right] & D & =1.380+0.000\ln\left[E\Pi_{1}\right]\\ WS & =27.497-0.0000\left[E\Pi_{1}\right] & WS & =85.069-0.468\ln\left[E\Pi_{1}\right]\\ MW & =310.144+0.0000\left[E\Pi_{1}\right] & MW & =57.531+2.177\ln\left[E\Pi_{1}\right]\\ VP & =1.312-0.0000\left[E\Pi_{1}\right] & VP & =1.005+0.002\ln\left[E\Pi_{1}\right]\\ & Quadratic\;Models: & \\ MP & =189.318+0.0000\left[E\Pi_{1}\right]+0.0000{\left[E\Pi_{1}\right]}^{2} & \\ BP & =497.015+0.0000\left[E\Pi_{1}\right]+0.0000{\left[E\Pi_{1}\right]}^{2} & \\ D & =1.409-0.0000\left[E\Pi_{1}\right]+0.0000{\left[E\Pi_{1}\right]}^{2} & \\ WS & =27.497-0.0000\left[E\Pi_{1}\right]+0.0000{\left[E\Pi_{1}\right]}^{2} & \\ MW & =310.144+0.0000\left[E\Pi_{1}\right]+0.0000{\left[E\Pi_{1}\right]}^{2} & \\ VP & =1.312-0.0000\left[E\Pi_{1}\right]+0.0000{\left[E\Pi_{1}\right]}^{2} & \end{array}$$

### 4.3 Multiplicative second exponential Zagreb index $E\Pi_{2}\left(G\right)$

$$\begin{array}{llll} & Linear\;Models: & & Logarithmic\;Models:\\ MP & =187.936+0.0000\left[E\Pi_{2}\right] & MP & =165.263+0.161\ln\left[E\Pi_{2}\right]\\ BP & =512.888+0.0000\left[E\Pi_{2}\right] & BP & =138.776+2.599\ln\left[E\Pi_{2}\right]\\ D & =1.383+0.0000\left[E\Pi_{2}\right] & D & =1.372+0.000\ln\left[E\Pi_{2}\right]\\ WS & =24.739+0.0000\left[E\Pi_{2}\right] & WS & =80.883-0.360\ln\left[E\Pi_{2}\right]\\ MW & =321.394+0.0000\left[E\Pi_{2}\right] & MW & =77.152+1.673\ln\left[E\Pi_{2}\right]\\ VP & =1.293-0.0000\left[E\Pi_{2}\right] & VP & =1.031+0.001\ln\left[E\Pi_{2}\right]\\ & Quadratic\;Models: & \\ MP & =0.0000+0.0000\left[E\Pi_{2}\right]+0.0000{\left[E\Pi_{2}\right]}^{2} & \\ BP & =0.0000+0.0000\left[E\Pi_{2}\right]+0.0000{\left[E\Pi_{2}\right]}^{2} & \\ D & =0.0000+0.0000\left[E\Pi_{2}\right]+0.0000{\left[E\Pi_{2}\right]}^{2} & \\ WS & =0.0000+0.0000\left[E\Pi_{2}\right]+0.0000{\left[E\Pi_{2}\right]}^{2} & \\ MW & =0.0000+0.0000\left[E\Pi_{2}\right]+0.0000{\left[E\Pi_{2}\right]}^{2} & \\ VP & =0.0000+0.0000\left[E\Pi_{2}\right]+0.0000{\left[E\Pi_{2}\right]}^{2} & \end{array}$$

### 4.4 Geometric arithmetic index GA(G)

$$\begin{array}{llll} & Linear\;Models: & & Logarithmic\;Models:\\ MP & =163.841+1.081\left[GA\right] & MP & =122.256+22.275\ln\left[GA\right]\\ BP & =116.656+17.429\left[GA\right] & BP & =-380.351+301.831\ln\left[GA\right]\\ D & =1.404+0.000\left[GA\right] & D & =1.221+0.058\ln\left[GA\right]\\ WS & =84.592-2.440\left[GA\right] & WS & =183.946-52.099\ln\left[GA\right]\\ MW & =56.994+11.469\left[GA\right] & MW & =-270.249+198.679\ln\left[GA\right]\\ VP & =1.086+0.006\left[GA\right] & VP & =0.548+0.225\ln\left[GA\left(G\right)\right]\\ & Quadratic\;Models: & \\ MP & =138.370+4.054\left[GA\right]-0.066{\left[GA\right]}^{2} & \\ BP & =223.976+4.901\left[GA\right]+0.278{\left[GA\right]}^{2} & \\ D & =0.953+0.052\left[GA\right]-0.001{\left[GA\right]}^{2} & \\ WS & =185.134-14.176\left[GA\right]+0.261{\left[GA\right]}^{2} & \\ MW & =97.890+6.695\left[GA\right]+0.106{\left[GA\right]}^{2} & \\ VP & =-0.454+0.186\left[GA\right]-0.004{\left[GA\right]}^{2} & \end{array}$$

### 4.5 Multiplicative geometric arithmetic index GAΠ(G)

$$\begin{array}{llll} & Linear\;Models: & & Logarithmic\;Models:\\ MP & =180.561+0.004\left[GA\Pi \right] & MP & =150.013+6.506\ln\left[GA\Pi \right]\\ BP & =413.314+0.054\left[GA\Pi \right] & BP & =105.714+70.086\ln\left[GA\Pi \right]\\ D & =1.395+0.0000\left[GA\Pi \right] & D & =1.265+0.022\ln\left[GA\Pi \right]\\ WS & =31.858-0.002\left[GA\Pi \right] & WS & =91.648-10.717\ln\left[GA\Pi \right]\\ MW & =258.505+0.033\left[GA\Pi \right] & MW & =53.998+45.428\ln\left[GA\Pi \right]\\ VP & =1.405-0.0000\left[GA\Pi \right] & VP & =1.078+0.024\ln\left[GA\Pi \right]\\ & Quadratic\;Models: & \\ MP & =172.688+0.017\left[GA\Pi \right]-0.0000{\left[GA\Pi \right]}^{2} & \\ BP & =347.333+0.166\left[GA\Pi \right]-0.0000{\left[GA\Pi \right]}^{2} & \\ D & =1.417-0.0000\left[GA\Pi \right]+0.0000{\left[GA\Pi \right]}^{2} & \\ WS & =53.368-0.039\left[GA\Pi \right]+0.0000{\left[GA\Pi \right]}^{2} & \\ MW & =203.663+0.126\left[GA\Pi \right]-0.0000{\left[GA\Pi \right]}^{2} & \\ VP & =1.148+0.0000\left[GA\Pi \right]-0.0000{\left[GA\Pi \right]}^{2} & \end{array}$$

### 4.6 Symmetric division degree index SDD(G)

$$\begin{array}{llll} & Linear\;Models: & & Logarithmic\;Models:\\ MP & =165.972+0.384\left[SDD\right] & MP & =99.804+22.375\ln\left[SDD\right]\\ BP & =43.587+7.949\left[SDD\right] & BP & =-1111.166+409.476\ln\left[SDD\right]\\ D & =1.350+0.001\left[SDD\right] & D & =1.026+0.092\ln\left[SDD\right]\\ WS & =88.124-1.004\left[SDD\right] & WS & =288.543-65.310\ln\left[SDD\right]\\ MW & =25.384+4.962\left[SDD\right] & MW & =-688.753+253.950\ln\left[SDD\right]\\ VP & =0.989+0.004\left[SDD\right] & VP & =-0.235+0.364\ln\left[SDD\right]\\ & Quadratic\;Models: & \\ MP & =134.066+1.684\left[SDD\right]-0.011{\left[SDD\right]}^{2} & \\ BP & =4.032+9.560\left[SDD\right]-0.013{\left[SDD\right]}^{2} & \\ D & =0.814+0.023\left[SDD\right]+0.000{\left[SDD\right]}^{2} & \\ WS & =265.783-8.241\left[SDD\right]+0.060{\left[SDD\right]}^{2} & \\ MW & =-0.979+6.036\left[SDD\right]-0.009{\left[SDD\right]}^{2} & \\ VP & =-1.661+0.112\left[SDD\right]-0.001{\left[SDD\right]}^{2} & \end{array}$$

## 5 Results and discussions

In this section, we delve into the statistical analysis of our regression models, which aimed to predict the anti-cancer properties of non-cancer medications based on their molecular characteristics represented by topological indices. The regression parameters for linear, quadratic, and logarithmic models were computed, providing insights into the relationships between the independent variables (topological indices) and the dependent variables (anti-cancer properties) The key parameters analyzed include the sample size (N), constant or *Y*-intercept (A), coefficients of the independent variables (b and c), correlation coefficient (r), and the percentage of variation explained by the linear model $\left(r^{2}\right)$ . These parameters are crucial for understanding the predictive accuracy and significance of the regression models.

The correlation coefficient (r) indicates the strength and direction of the relationship between variables, with values ranging from −1 to +1. A positive coefficient signifies a direct relationship, while a negative coefficient suggests an inverse relationship. The high correlation coefficients observed in our analysis indicate strong associations between the topological indices and anti-cancer properties, supporting the validity of our models. Moreover, the *p*-values associated with each term in the regression models were examined to test the null hypothesis that the coefficient is zero, implying no effect. A smaller *p*-value suggests that changes in the predictor variables are associated with changes in the response variable, indicating the significance of the predictors. In our analysis, all *p*-values were found to be zero, indicating the statistical significance of the regression models. Furthermore, we conducted additional tests to assess the overall predictive capability of our models. The F-value resulting from these tests helps to determine whether the model has predictive power beyond chance. The significant F-values obtained in our analysis confirm the predictive capability of our regression models in accurately predicting anti-cancer properties based on topological indices. These statistical characteristics for logarithmic, linear, and quadratic QSPR models for various topological indices are presented in Tables 5–10. Overall, the statistical analysis presented in this section underscores the robustness and reliability of our regression models in identifying non-cancer medications with potential anti-cancer properties. These findings provide a solid foundation for further exploration and validation of drug repurposing strategies in cancer therapy.

**TABLE 5:** Statistical parameters for ABC-R(G).

Properties	N	A	b	c	r	$r^{2}$	F	P
Linear Regression Model								
MP	15	165.97	3.277	—	0.254	0.065	0.897	0.361
BP	15	96.074	60.496	—	0.820	0.672	26.648	0.000
D	15	1.352	0.006	—	0.077	0.006	0.078	0.784
WS	15	86.975	−8.398	—	0.365	0.133	1.992	0.182
MW	15	50.608	38.815	—	0.975	0.951	249.667	0.000
VP	15	0.984	0.034	—	0.115	0.013	0.173	0.684
Quadratic Regression Model								
MP	15	98.874	59.410	0.081	0.820	0.672	12.300	0.001
BP	15	138.290	14.016	−0.798	0.297	0.088	0.579	0.575
D	15	0.892	0.185	−0.13	0.407	0.166	1.192	0.337
WS	15	223.388	−61.303	3.929	0.558	0.311	2.706	0.107
MW	15	67.781	32.155	0.495	0.975	0.951	117.590	0.000
VP	15	0.928	0.775	−0.055	0.477	0.228	1.770	0.212
Logarithmic Regression Model								
MP	15	151.661	20.670	—	0.289	0.083	1.183	0.296
BP	15	−70.118	328.130	—	0.801	0.642	23.307	0.000
D	15	1.230	0.091	—	0.201	0.040	0.545	0.473
WS	15	132.282	−57.665	—	0.451	0.203	3.319	0.092
MW	15	−50.421	207.482	—	0.939	0.882	96.894	0.000
VP	15	0.640	0.320	—	0.196	0.038	0.519	0.484

**TABLE 6:** Statistical parameters for $E\Pi_{1}$ (G).

Properties	N	A	b	c	r	$r^{2}$	F	P
Linear Regression Model								
MP	15	189.318	0.000	—	0.23	0.001	0.007	0.935
BP	15	497.015	0.000	—	0.488	0.238	4.070	0.065
D	15	1.409	−0.000	—	0.150	0.022	0.298	0.595
WS	15	27.497	−0.000	—	0.048	0.002	0.030	0.866
MW	15	310.144	0.000	—	0.522	0.273	4.869	0.46
VP	15	1.312	−0.000	—	0.297	0.088	1.259	0.282
Quadratic Regression Model								
MP	15	189.318	0.000	0.000	0.023	0.001	0.003	0.997
BP	15	497.015	0.000	0.000	0.488	0.238	1.879	0.195
D	15	1.409	−0.000	0.000	0.150	0.022	0.137	0.873
WS	15	27.497	−0.000	0.000	0.048	0.002	0.014	0.986
MW	15	310.144	0.000	0.000	0.522	0.273	2.247	0.148
VP	15	1.312	−0.000	0.000	0.297	0.088	0.581	0.574
Logarithmic Regression Model								
MP	15	165.206	0.195	—	0.270	0.073	1.020	0.331
BP	15	104.723	3.411	—	0.826	0.682	27.899	0.000
D	15	1.380	0.000	—	0.030	0.001	0.012	0.914
WS	15	85.069	−0.468	—	0.363	0.132	1.971	0.184
MW	15	57.531	2.177	—	0.977	0.955	275.505	0.000
VP	15	1.005	0.002	—	0.107	0.012	0.152	0.703

**TABLE 7:** Statistical parameters for $E\Pi_{2}$ (G).

Properties	N	A	b	c	r	$r^{2}$	F	P
Linear Regression Model								
MP	15	187.936	0.000	—	0.151	0.023	0.301	0.592
BP	15	512.888	0.000	—	0.305	0.093	1.331	0.269
D	15	1.383	0.000	—	0.205	0.042	0.571	0.463
WS	15	24.739	0.000	—	0.085	0.007	0.094	0.764
MW	15	321.394	0.000	—	0.262	0.068	0.955	0.346
VP	15	1.293	−0.000	—	0.262	0.068	0.955	0.346
Quadratic Regression Model								
MP	15	0.000	0.000	0.000	1.000	1.000	0.000	0.000
BP	15	0.000	0.000	0.000	1.000	1.000	0.000	0.000
D	15	0.000	0.000	0.000	1.000	1.000	0.000	0.000
WS	15	0.000	0.000	0.000	1.000	1.000	0.000	0.000
MW	15	0.000	0.000	0.000	1.000	1.000	0.000	0.000
VP	15	0.000	0.000	0.000	1.000	1.000	0.000	0.000
Logarithmic Regression Model								
MP	15	165.263	0.161	—	0.286	0.082	1.156	0.302
BP	15	138.776	2.599	—	0.807	0.651	24.280	0.000
D	15	1.372	0.000	—	0.046	0.002	0.028	0.870
WS	15	80.883	−0.360	—	0.358	0.18	1.908	0.190
MW	15	77.152	1.673	—	0.963	0.927	165.807	0.000
VP	15	1.031	0.001	—	0.101	0.010	0.133	0.721

**TABLE 8:** Statistical parameters for GA(G).

Properties	N	A	b	c	r	$r^{2}$	F	P
Linear Regression Model								
MP	15	163.841	1.081	—	0.285	0.081	1.146	0.304
BP	15	116.656	17.429	—	0.802	0.643	23.462	0.000
D	15	1.404	0.000	—	0.011	0.000	0.002	0.968
WS	15	84.592	−2.440	—	0.360	0.129	1.931	0.188
MW	15	56.994	11.469	—	0.978	0.957	290.228	0.000
VP	15	1.086	0.006	—	0.068	0.005	0.061	0.809
Quadratic Regression Model								
MP	15	138.370	4.054	−0.066	0.322	0.104	0.694	0.519
BP	15	223.976	4.901	0.278	0.810	0.656	11.430	0.002
D	15	0.953	0.052	−0.001	0.420	0.176	1.286	0.312
WS	15	185.134	−14.176	0.261	0.490	0.240	1.895	0.193
MW	15	97.890	6.695	0.106	0.981	0.963	157.319	0.000
VP	15	−0.454	0.186	−0.004	0.405	0.164	1.179	0.341
Logarithmic Regression Model								
MP	15	122.256	22.275	—	0.322	0.104	1.501	0.242
BP	15	−380.351	301.831	—	0.762	0.581	17.994	0.001
D	15	1.221	0.058	—	0.133	0.018	0.233	0.637
WS	15	183.946	−52.099	—	0.421	0.177	2.804	0.118
MW	15	−270.249	198.679	—	0.930	0.864	82.682	0.000
VP	15	0.548	0.225	—	0.142	0.020	0.269	0.613

**TABLE 9:** Statistical parameters for GAΠ (G).

Properties	N	A	b	c	r	$r^{2}$	F	P
Linear Regression Model								
MP	15	180.561	0.004	—	0.294	0.087	1.232	0.287
BP	15	413.314	0.054	—	0.676	0.457	10.946	0.006
D	15	1.395	0.000	—	0.013	0.000	0.002	0.963
WS	15	31.858	−0.002	—	0.099	0.010	0.129	0.726
MW	15	258.505	0.033	—	0.758	0.575	17.578	0.001
VP	15	1.405	−0.000	—	0.254	0.065	0.899	0.360
Quadratic Regression Model								
MP	15	172.688	0.017	−0.000	0.361	0.130	0.899	0.433
BP	15	347.333	0.166	−0.000	0.742	0.551	7.361	0.008
D	15	1.417	−0.000	0.000	0.092	0.009	0.052	0.950
WS	15	53.368	−0.039	0.000	0.335	0.112	0.757	0.490
MW	15	203.663	0.126	−0.000	0.893	0.798	23.636	0.000
VP	15	1.148	0.000	−0.000	0.393	0.154	1.093	0.386
Logarithmic Regression Model								
MP	15	150.013	6.506	—	0.396	0.157	2.417	0.144
BP	15	105.714	70.086	—	0.745	0.556	16.259	0.001
D	15	1.265	0.022	—	0.209	0.044	0.593	0.455
WS	15	91.648	−10.717	—	0.365	0.133	1.999	0.181
MW	15	53.998	45.428	—	0.896	0.802	52.653	0.000
VP	15	1.078	0.024	—	0.065	0.004	0.056	0.817

**TABLE 10:** Statistical parameters for SDD(G).

Properties	N	A	b	c	r	$r^{2}$	F	P
Linear Regression Mode								
MP	15	165.972	0.384	—	0.234	0.055	0.754	0.401
BP	15	43.587	7.949	—	0.846	0.716	32.752	0.000
D	15	1.350	0.001	—	0.074	0.006	0.072	0.793
WS	15	88.124	−1.004	—	0.342	0.117	1.724	0.212
MW	15	25.384	4.962	—	0.979	0.959	300.379	0.000
VP	15	0.989	0.004	—	0.103	0.011	0.140	0.715
Quadratic Regression Model								
MP	15	134.066	1.684	−0.011	0.272	0.074	0.480	0.630
BP	15	4.032	9.560	−0.013	0.847	0.717	15.184	0.001
D	15	0.814	0.023	0.000	0.374	0.140	0.979	0.0404
WS	15	265.783	−8.241	0.060	0.552	0.304	2.625	0.113
MW	15	−0.979	6.036	−0.009	0.980	0.960	143.618	0.000
VP	15	−1.661	0.112	−0.001	0.516	0.267	2.181	0.156
Logarithmic Regression Model								
MP	15	99.804	22.375	—	0.262	0.069	0.961	0.345
BP	15	−1111.166	409.476	—	0.839	0.704	30.923	0.000
D	15	1.026	0.092	—	0.171	0.029	0.389	0.543
WS	15	288.543	−65.310	—	0.429	0.184	2.926	0.111
MW	15	−688.753	253.950	—	0.964	0.930	173.293	0.000
VP	15	−0.235	0.364	—	0.187	0.035	0.473	0.504

The study emphasizes the importance of topological descriptors in predicting the molecular structures of anti-cancer drugs, crucial for estimating the molecular weight of non-cancer medications. Quantitative Structure-Property Relationship (QSPR) methodology stands out as a robust approach utilized across diverse fields such as drug design, material science, environmental chemistry, cheminformatics, and computational chemistry. QSPR methodology is focused on establishing mathematical relationships between the chemical structure of compounds and their properties, enabling the prediction of properties based on molecular characteristics. Unlike QSAR, which typically addresses biological activities, QSPR specifically targets the physical and chemical properties of compounds. Molecular modeling techniques often entail complex simulations and calculations to forecast molecular behavior accurately. Ultimately, QSPR methodology provides a systematic and quantitative means to predict physicochemical properties of chemical compounds based on their molecular structure, distinguishing it from other methodologies like QSAR, MD simulation, DFT, machine learning models, and hybrid QSAR/QSPR models, each offering its unique advantages and limitations depending on the specific research objectives and applications.

## 6 Conclusion

This study demonstrates the efficacy of utilizing Quantitative Structure-Property Relationship (QSPR) modeling, specifically leveraging topological indices, to identify non-cancer medications with potential anti-cancer properties. The systematic approach outlined ranging from data collection and molecular descriptor calculation to model construction and experimental validation has proven to be a robust framework for drug repurposing. By focusing on topological indices, we have highlighted the significant role that molecular structure plays in determining pharmacological interactions relevant to anti-cancer activity.

Our findings indicate that several non-cancer drugs, identified through our QSPR models, exhibit promising anti-cancer properties, warranting further experimental validation and clinical trials. The use of topological indices in the modeling process has provided critical insights into the structural attributes that contribute to anti-cancer efficacy, thus enhancing our understanding of drug action mechanisms.

The integration of computational modeling with experimental validation offers a cost-effective and accelerated pathway for expanding the repertoire of anti-cancer agents. This research not only underscores the potential of drug repurposing strategies in oncology but also establishes a foundation for future studies to explore and refine the application of QSPR models. Ultimately, this approach holds the promise of improving therapeutic outcomes for cancer patients by identifying new uses for existing medications, thereby bridging the gap between drug discovery and clinical application.

## Data Availability

The raw data supporting the conclusions of this article will be made available by the authors, without undue reservation.
